# The Gut–Heart Axis: Molecular Perspectives and Implications for Myocardial Infarction

**DOI:** 10.3390/ijms252212465

**Published:** 2024-11-20

**Authors:** Katherine Rivera, Leticia Gonzalez, Liena Bravo, Laura Manjarres, Marcelo E. Andia

**Affiliations:** 1 Doctoral Program in Medical Sciences, Faculty of Medicine, Pontificia Universidad Católica de Chile, Santiago de Chile 8331010, Chile; ksrivera@uc.cl; 2 Biomedical Imaging Center, School of Medicine, Pontificia Universidad Católica de Chile, Santiago de Chile 7820436, Chile; 3 Millennium Institute for Intelligent Healthcare Engineering iHEALTH, Santiago de Chile 7820436, Chile

**Keywords:** myocardial infarction, gut–heart axis, gut microbiota, intestinal barrier, gut metabolites

## Abstract

Myocardial infarction (MI) remains the leading cause of death globally, imposing a significant burden on healthcare systems and patients. The gut–heart axis, a bidirectional network connecting gut health to cardiovascular outcomes, has recently emerged as a critical factor in MI pathophysiology. Disruptions in this axis, including gut dysbiosis and compromised intestinal barrier integrity, lead to systemic inflammation driven by gut-derived metabolites like lipopolysaccharides (LPSs) and trimethylamine N-oxide (TMAO), both of which exacerbate MI progression. In contrast, metabolites such as short-chain fatty acids (SCFAs) from a balanced microbiota exhibit protective effects against cardiac damage. This review examines the molecular mediators of the gut–heart axis, considering the role of factors like sex-specific hormones, aging, diet, physical activity, and alcohol consumption on gut health and MI outcomes. Additionally, we highlight therapeutic approaches, including dietary interventions, personalized probiotics, and exercise regimens. Addressing the gut–heart axis holds promise for reducing MI risk and improving recovery, positioning it as a novel target in cardiovascular therapy.

## 1. Introduction

Myocardial infarction (MI), commonly known as heart attack, is the leading cause of death worldwide, significantly contributing to both morbidity and healthcare costs [1]. MI typically results from a completely or partially obstructed coronary blood flow, leading to ischemia and myocardial damage that can culminate in heart failure (HF) and increased mortality among survivors [2,3]. Despite advancements in diagnostic techniques, pharmacological therapies, and surgical interventions, the prevalence and mortality of MI continue to rise, underscoring the need for novel therapeutic approaches that address underlying and exacerbating mechanisms [4].

Traditionally, MI pathophysiology has focused on coronary artery disease (CAD) and its established traditional cardiac risk factors, such as dyslipidemia, hypertension, smoking, diabetes mellitus, and obesity [5]. However, emerging research has highlighted the gut–heart axis as a significant contributor to cardiovascular outcomes, challenging the traditional view of the gut as an isolated organ system [6,7]. This axis represents a dynamic, bidirectional network in which gut health, specifically through intestinal barrier integrity and gut microbiota homeostasis, plays a pivotal role in maintaining immune and metabolic balance in the host [8,9,10]. Given these findings, the gut–heart axis is increasingly recognized as a central component of cardiovascular health, linking extrinsic factors such as diet, exercise, age, and sex hormones to MI outcomes. This review comprehensively analyzes the molecular mediators and signaling pathways that constitute the gut–heart axis. By examining how integrity of the gut barrier, microbiota composition, and systemic inflammation influence MI, we highlight innovative therapeutic strategies that target this axis, such as dietary interventions, probiotics, and exercise, to mitigate the risk of MI and improve recovery outcomes. These insights advance our understanding of the gut–heart axis as a promising target in cardiovascular therapeutics.

## 2. The Gut–Heart Axis: A Molecular Perspective

### 2.1. Intestinal Barrier Integrity and MI: Mechanistic Insights

**Figure 1:**
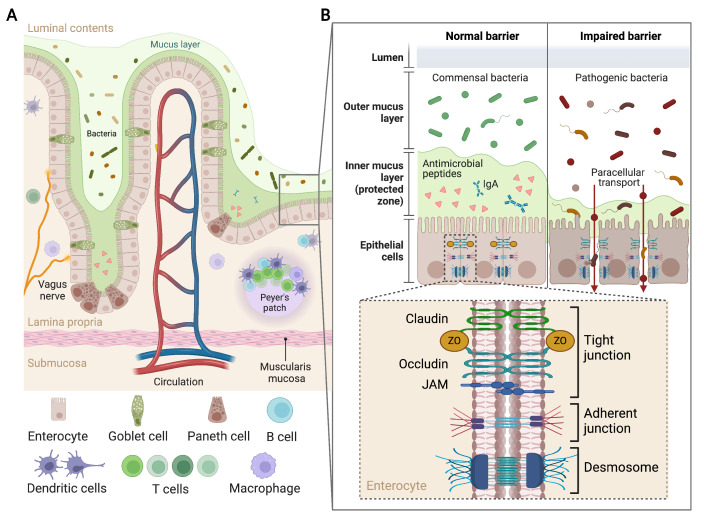
Host–microorganism interface. (**A**) Schematic representation of the main components of the intestinal barrier. (**B**) Junctional complexes linking adjacent epithelial cells in normal and impaired intestinal barrier.

The intestinal barrier serves as a critical interface between the external environment and the internal systems of the body, maintaining physiological homeostasis by selectively absorbing nutrients while blocking harmful antigens and microorganisms from entering the systemic circulation (Figure 1) [11]. Structurally, the intestinal barrier consists of several layers that comprise chemical, immunological, physical, and microbial components, which together regulate its structural integrity and metabolic activity (Figure 1A). The mucus secreted by goblet cells forms the chemical shield rich in antimicrobial peptides and immunoglobulin A (IgA), which allows the growth of commensal bacteria, limiting pathogenic bacteria proliferation and enhancing immunological defense [12]. The physical barrier of the gut is formed by a single layer of enterocytes connected by tight junction proteins (e.g., claudins, occluding, zonula occludens (ZOs), and the junctional adhesion molecule (JAM)), along with adherent junctions and desmosomes (Figure 1B). Together, these structures are essential for maintaining gut integrity by regulating paracellular permeability, thereby preventing luminal antigens from entering the underlying tissue and triggering chronic inflammation [13,14].

Disruptions in the components of the gut barrier are associated with various intestinal and systemic diseases [13,15,16,17,18]. In particular, intestinal dysfunction has been closely linked to cardiovascular diseases (CVDs), with intestinal-related conditions increasing the risk of atherosclerosis, CAD, stroke, HE, and atrial fibrillation [19]. Several studies have established a connection between MI and intestinal barrier disruptions (Table 1).

For example, elevated serum levels of ZO, a critical tight junction protein responsible for maintaining intestinal barrier integrity, have been observed in MI patients and positively correlate with systemic inflammation markers such as IL-10 [20,21,22,23]. Additionally, intestinal fatty acid-binding protein (I-FABP), a marker of gut mucosal injury, has recently been linked to larger infarct sizes and poorer cardiac function in acute MI [24]. This highlights its value as a biomarker for MI severity and enables early identification of patients at higher risk of complications [24]. Another significant marker of compromised gut integrity is serum lipopolysaccharide (LPS), a bacterial-derived endotoxin significantly elevated in ST-elevation MI (STEMI) patients, which positively correlates with systemic inflammation, worsened cardiac damage, and larger post-MI infarct sizes [20,21,22,23,25,26]. Also, elevated serum LPS levels were significantly correlated with serum ZO and blood bacterial load, raising the possibility that intestinal bacteria might invade the blood post-MI as a consequence of enhanced gut permeability [21,23]. Furthermore, elevated levels of LPS are associated with both short- and long-term risk of cardiovascular events in patients with community-acquired pneumonia, suggesting that ZO could serve as a useful biomarker for identifying individuals at greater risk of adverse ischemic outcomes [27]. LPS typically translocates from the gut lumen into circulation when intestinal barrier integrity is compromised, leading to the activation of toll-like receptor 4 (TLR4) on immune cells [21,25]. This activation triggers a pro-inflammatory cascade, resulting in cytokine release that can exacerbate myocardial damage and impair recovery post-MI. Supporting this connection, immunohistochemical analysis of coronary thrombi in MI patients has revealed the presence of LPS within the thrombus, along with the overexpression of TLR4 and cathepsin G in leukocytes, which promotes leukocyte–platelet binding and activation [21,28]. These findings suggest a mechanistic link between gut-derived endotoxins and thrombotic processes in MI. Notably, injections of E. coli-LPS in mice, which archived LPS levels similar to those found in human coronary thrombi, were associated with increased arterial thrombosis and platelet activation [21]. This evidence indicates that LPS not only drives systemic inflammation but also promotes thrombogenesis in MI, as gut-derived LPS exacerbates MI severity by fostering pro-thrombotic conditions and intensifying inflammation within coronary vessels. Furthermore, circulating D-lactate—a byproduct of bacterial metabolism—is elevated in MI patients, correlating with systemic inflammation and an increased risk of post-MI HF, underscoring the critical role of gut barrier dysfunction in MI progression [26]. Experimental models further support these findings, demonstrating that disrupted gut integrity and microbial metabolites exacerbate inflammation, oxidative stress, and MI severity [21,23,26,29,30]. Emerging therapeutic strategies are now focusing on restoring gut barrier integrity, particularly by targeting pathways such as TLR4-mediated signaling. TLR4 inhibitors are currently under investigation for their potential to reduce LPS-induced inflammation and protect myocardial tissue after MI [21,31,32]. Preserving gut barrier function shows significant promise as a therapeutic approach in MI, although it remains unclear whether vascular or intestinal dysfunction is the primary trigger in this complex process [33].

### 2.2. Gut Microbiota Composition and MI

**Figure 2:**
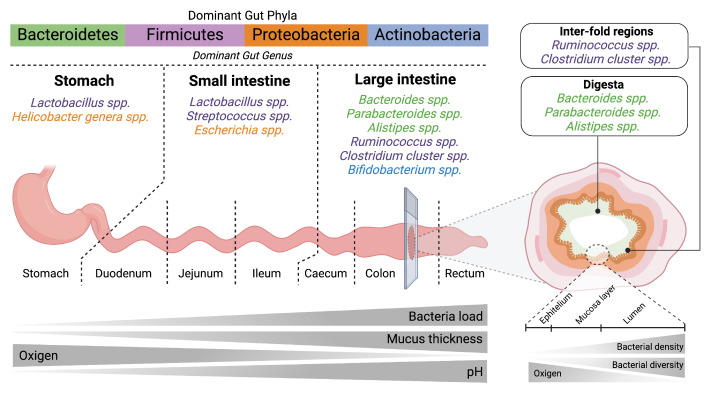
Complexity of the gut microbiota and its adaptation to different microenvironments in the lower GI tract. Four major bacterial phyla (Bacteroidetes, Firmicutes, Proteobacteria, and Actinobacteria) are found in different sections of the GI tract. Oxygen levels decrease progressively from the stomach to the colon, reflecting a shift from an aerobic to an anaerobic environment. Population density and mucus thickness both increase from the stomach to the colon, corresponding with higher microbial diversity and density in the large intestine, while pH decreases along the tract, providing favorable conditions for specific bacterial communities in different regions.

The gut microbiota is a complex ecosystem of trillions of symbiotic microorganisms, including bacteria, archaea, viruses, and fungi, which have coevolved with humans to support essential physiological functions [34]. Comprising approximately 70% of the body’s microbial cells, this community plays a key role in digestion, nutrient absorption, vitamin synthesis, and maintaining intestinal barrier integrity. It also produces crucial metabolites, such as short-chain fatty acids (SCFAs), which are vital for health [35,36]. The gut microbiota is predominantly made up of the bacterial phyla Firmicutes and Bacteroidetes, with significant diversity in composition and distribution throughout the gastrointestinal (GI) tract, particularly in the densely populated cecum and colon (see Figure 2) [37,38,39].

Due to this complexity, researchers often use fecal samples as a proxy to estimate gut microbiota composition [40]. Emerging research demonstrates that gut microbiota composition plays a significant role in cardiovascular health. Dysbiosis—characterized by a reduction in beneficial microbial species and an overgrowth of pathogenic bacteria—has been increasingly linked to intestinal and extra-intestinal conditions, including obesity, diabetes, and CVD [41,42,43]. Evidence suggest that gut microbiota significantly influences the severity and outcomes of MI by affecting systemic inflammation, immune modulation, and metabolic pathways. Disbiotic shifts can promote disease progression through mechanisms such as microbial translocation, altered production of gut-derived metabolites, and increased leakage of metabolic waste, thereby activating systemic inflammation. Advanced sequencing techniques, such as 16S rRNA and metagenomic analysis, have enabled a precise mapping of microbiota diversity and its impact on MI outcomes [44,45]. For instance, MI patients often show reduced levels of beneficial Firmicutes species and increased levels of pro-inflammatory bacteria like Bacteroidetes and Verrucomicrobia [46,47]. This imbalance leads to elevated levels of pro-inflammatory metabolites, exacerbating myocardial injury [26,46,48]. Additionally, gut microbiota has been implicated in modulating neutrophil extracellular trap (NET) formation, shedding light on mechanisms of ischemia/reperfusion (I/R) injury in MI [23,49]. Specific bacterial species have also been linked to MI severity; for example, increased levels of *Streptococcus salivarius* and *Klebsiella pneumonia* are associated with more severe MI, while *Roseburia hominis*, an SCFA producer, is inversely related to MI severity [50]. These findings underscore the potential of targeting gut microbiota for novel therapeutic strategies to mitigate MI risk and improve patient outcomes.

Metagenomic studies reveal significant microbial translocation into the systemic circulation following STEMI, which amplifies inflammation and increases the risk of subsequent cardiovascular events [26]. Pathogens like *Enterobacteriaceae* and *Escherichia coli* contribute to this process by releasing LPS and other factors that activate harmful immune responses in cardiac tissue [26,46,48]. Additionally, blood microbiota composition post-MI is influenced by low-density lipoprotein (LDL) levels, with elevated LDL promoting increased microbial diversity and inflammation [51]. Experimental models further support these findings. Suppressing gut microbiota in MI models reduced monocyte infiltration in the peri-infarct region, decreased ventricular rupture, and lowered mortality rates. Conversely, fecal microbiota transplantation from healthy donors minimizes infarct size and improved survival outcomes [30,52,53]. Interestingly, recent studies indicate that the role of the gut microbiota in MI pathogenesis may differ between diabetic and non-diabetic individuals due to variations in host metabolic states and microbial composition [54,55]. Diabetic MI patients typically exhibit an increased abundance of Firmicutes and a reduction in Bacteroidetes, alterations linked to systemic inflammation and metabolic dysregulation, which may contribute to a poorer prognosis. In contrast, non-diabetic MI patients show higher levels of beneficial bacteria, potentially explaining their relatively better cardiac outcomes following MI. These findings underscore a complex relationship between gut dysbiosis and MI, suggesting that targeted therapies focusing on the gut–heart axis could enhance MI recovery and reduce post-infarction complications. By understanding the differential impact of gut microbiota on MI progression in diabetic versus non-diabetic patients, future research can better tailor interventions to optimize cardiovascular outcomes in these distinct patient groups.

### 2.3. Molecular Pathways Linking Gut-Derived Metabolites to MI

The gut microbiota converts dietary nutrients into bioactive metabolites that significantly influence host physiology [10]. The balance between harmful and beneficial metabolites can greatly impact MI outcomes. One such metabolite is trimethylamine (TMA), which is produced by intestinal bacteria from dietary sources like choline, betaine, and L-carnitine. TMA is subsequently oxidized in the liver to form trimethylamine-N-oxide (TMAO), which has been consistently linked to an increased risk of adverse cardiovascular outcomes, including MI and mortality [56,57]. As such, TMAO is emerging as a potential biomarker for assessing cardiovascular risk and guiding therapeutic interventions. Studies have shown that elevated TMAO levels can predict a heightened risk of major adverse cardiovascular events, even after adjustment for traditional risk factors [58]. Furthermore, high circulating TMAO levels are associated with a poorer prognosis post-MI, correlating with an increased risk of death and recurrent infarctions during follow-up periods [59,60,61,62,63,64,65,66]. While the precise mechanisms by which TMAO increases MI risk are not yet fully elucidated, current evidence suggests that TMAO induces alterations in cholesterol metabolism, promotes inflammation, oxidative stress, and endothelial dysfunction—all key factors in vascular dysfunction [67,68,69,70,71,72]. Additionally, TMAO contributes to plaque instability and rupture, enhancing platelet aggregation and thereby increasing the risk of thrombosis, a critical factor in MI [73,74,75,76]. Emerging evidence also indicates that TMAO promotes myocardial hypertrophy, exacerbates myocardial interstitial and perivascular fibrosis, and impairs cardiac compliance and function, collectively hindering post-MI recovery [29,64,77,78,79,80]. These findings underscore the critical role of TMAO in worsening cardiovascular outcomes, suggesting that interventions targeting TMAO pathways may improve MI prognosis.

On the other hand, SCFAs such as butyrate, acetate, malonate, succinate, and propionate are produced by anaerobic bacteria through the fermentation of dietary fiber [81,82]. Unlike TMAO, SCFAs generally exhibit cardioprotective effects that positively influence MI outcomes. Several studies have shown that reduced SCFA levels impair immune response and increase mortality after MI, suggesting a protective role for SCFA in cardiac health [53,83,84,85,86]. Butyrate and propionate, in particular, have been shown to reduce apoptosis and enhance cell survival in experimental models, potentially preventing MI [87]. Butyrate also mitigates myocardial fibrosis by regulating macrophages M1/M2 polarization and promoting mitochondrial function recovery [88,89,90]. Propionate has been shown to lower the risk of MI-induced ventricular arrhythmias and improve cardiac electrophysiology stability, partially through parasympathetic activation [91]. Similarly, dietary interventions that enhance SCFA production emphasize their potential as therapeutic targets to reduce ischemic injury and systemic inflammation [92,93]. However, recent evidence suggests that the cardioprotective effects of SCFAs are context-dependent, influenced by specific G-protein-coupled receptors they activate [94]. For instance, SCFAs activate free fatty acid receptor 2 (FFAR2) to trigger anti-inflammatory pathways, whereas activation of FFAR3 may stimulate sympathetic nervous activity and adrenal catecholamine release. This activation can increase heart rate, blood pressure, and pro-inflammatory cytokine levels, potentially exacerbating cardiac stress [95,96,97,98]. Thus, optimizing therapeutic strategies using SCFAs requires careful consideration of their receptor-specific actions to maximize their benefits.

Beyond TMAO and SCFAs, other gut-derived metabolites, such as bile acids (BAs) and indole derivatives, play significant roles in cardiovascular health. Bile acids, which are modified by gut microbiota, influence lipid metabolism and systemic inflammation through receptors like FXR and TGR5. These pathways are strongly associated with the presence and severity of CAD, particularly in MI patients; however, the precise molecular mechanism remain controversial [99,100]. Also, indole derivatives, produced from microbial metabolism of tryptophan, exert anti-inflammatory effects by activating the aryl hydrocarbon receptor (AhR), thereby reducing endothelial dysfunction and vascular inflammation [101,102,103]. These pathways highlight additional therapeutic targets, suggesting that a broader range of gut-derived metabolites could be leveraged to mitigate MI risk.

## 3. Influence of Sex, Age, and Lifestyle Factors on the Gut–Heart Axis

**Figure 3:**
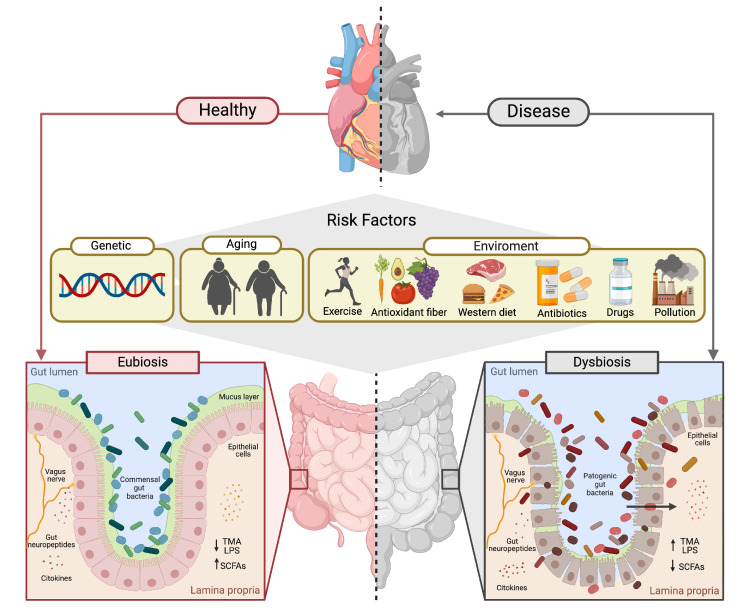
Risk factors in the gut–heart axis in health and disease. In a healthy state (eubiosis), factors like exercise and a fiber- and antioxidant-rich diets support beneficial gut bacteria, boosting SCFA production and limiting harmful compounds like TMA and LPS. Conversely, risk factors such as a Western diet, aging, antibiotics, and pollution lead to gut dysbiosis, where pathogenic bacteria increase inflammatory mediators, impair gut integrity, and raise systemic inflammation and MI risk.

The gut–heart axis is influenced by various host factors, with sex, age, and lifestyle playing pivotal roles in modulating gut microbiota composition and function. These factors not only affect microbiota diversity but also influence metabolite production, immune response, and inflammatory pathways, all of which are crucial for MI risk and recovery (Figure 3).

### 3.1. Sex-Related Differences

Health and disease susceptibility, including CVD, are influenced by sex chromosomes and hormone-driven physiological differences. Modifiable CVD risk factors—such as smoking, obesity, diabetes, and socioeconomic status—tend to have a stronger impact on women, who generally develop CVD later in life than men but experience more comorbidities and poorer outcomes after MI [104,105,106,107]. Diagnostic biomarkers, like cardiac troponin, also show lower sensitivity in women, highlighting the need for sex-specific cut-off values in clinical practice [108]. The increased CVD risk in postmenopausal women, driven by reduced estrogen levels, has led to studies demonstrating that hormone replacement can thereby reduce CVD risk, emphasizing the modulating effects of sex hormones throughout life [109,110,111]. Recent research shows that these hormonal effects extend to gut microbiota composition, potentially influencing MI risk. Animal studies indicate that gut microbiota profiles vary significantly by sex, with interventions like gonadectomy shifting microbiota composition towards profiles typically observed in the opposite sex [112,113]. In male mice, certain bacteria species are more prevalent, with sex accounting for 11.6% of microbiota variance [114]. In humans, women generally have lower levels of the Bacteroidetes phylum, while castration in male mice shifts their microbiota profiles to resemble those of females, a change reversible with testosterone supplementation [112,115,116]. Fecal transplantation and hormone studies further demonstrate sex-related microbiota differences [117,118,119,120]. Sex-specific differences in gut-derived metabolites also influence MI outcomes. For instance, MI patients initially have higher circulating TMAO levels in women than men, with levels normalizing through dietary changes and exercise during cardiac rehabilitation [121]. Moreover, distinct metabolomic profiles, such as elevated histidine and O-acetyl-glycoprotein (OAG) in men and succinate in women, underscore the importance of sex-specific biomarkers for improving MI risk stratification and treatment [122].

### 3.2. Age-Related Changes

The incidence of CVD increases significantly with age, primarily due to prolonged exposure to risk factors and cumulative physiological changes [123,124]. Early management of these risk factors can yield substantial benefits; for example, a 10% reduction in cholesterol levels in younger adults can decrease ischemic heart disease risk by up to 54%, compared to only 27% in older adults [125,126]. The incidence of MI also escalates with age, doubling in men and increasing fivefold in women between ages 55–64 and 85–94 [127]. Mortality rates post-MI are particularly high in elderly patients, partly due to delayed diagnosis from atypical symptoms and increased procedural complications [128,129]. Aging also significantly impacts gut microbiota composition, which in turn influences cardiovascular health [130]. Early-life factors like birth type and feeding shape microbiota profiles, while diet and lifestyle drive composition in adulthood [131]. Generally, Bacteroidetes are more prevalent in childhood but decline with age, whereas Firmicutes increase [132]. In the elderly, Bacteroidetes levels rise, while beneficial anaerobes like *Bifidobacterium* decrease, and pathogenic groups such as *Enterobacteria* increase, contributing to chronic low-grade inflammation, a key factor in age-related conditions, including CVD [133,134,135]. Animal studies support these findings, showing that transferring microbiota from aged donors to younger, germ-free recipients induces inflammation, activating inflammatory pathways like TLR2 [135]. Aging is also associated with elevated plasma levels of TMAO, which correlate with adverse cardiovascular outcomes [136,137]. Higher TMAO levels in older adults result from a greater abundance of TMA-producing bacteria and are linked to markers of vascular aging, such as increased carotid intima–media thickness [122,138]. Additionally, elevated levels of TMAO and related metabolites (e.g., choline and carnitine) have been linked to higher mortality from both CVD and non-CVD causes in individuals over 65 [139]. These findings highlight the need for age-specific CVD treatment strategies. Approaches targeting TMAO reduction, gut microbiota modulation, and anti-inflammatory therapies may help mitigate age-related cardiovascular risks and improve outcomes in older adults.

### 3.3. Lifestyle Factors

#### 3.3.1. Unhealthy Diet

Poor diets, often high in refined grains, added sugars, salt, unhealthy fats, and animal-source foods, but low in whole grains, fruits, vegetables, legumes, fish, and nuts, are a major risk factor for CVD and contribute substantially to the global burden of non-communicable diseases [140,141]. Western diets, characterized by high saturated fats, refined carbohydrates, and processed foods, are closely linked to increased severity of coronary artery lesions and elevated risk of MI and stroke [142,143]. For example, frequent consumption of fried food is associated with a 28% increase in the risk of major cardiovascular events, partly because saturated and trans fats elevate cholesterol levels, promoting atherosclerosis and increasing MI risk [144,145,146]. Importantly, diets significantly affect gut microbiota, with dietary patterns accounting for 57% of microbiota variation, compared to just 12% from genetics [147]. High-fat diets, for instance, disrupt gut barrier integrity and promote endotoxemia by reducing protective bacteria [148]. A Western diet often leads to gut dysbiosis, marked by a decrease in beneficial Bacteroidetes and an increase in Firmicutes, which is linked to obesity and metabolic disturbances [149,150]. This imbalance reduces SCFA production and increases gut permeability, allowing endotoxins into circulation and promoting inflammation that drives atherosclerosis and MI [149,151]. Beyond saturated fats and refined carbs, other dietary patterns also influence MI risk and gut microbiota. High-salt diets are linked to hypertension, a major CVD risk factor, and reduced levels of beneficial *Lactobacillus* species, while low-fiber and polyphenol-poor diets fail to support SCFA-producing bacteria essential for gut health and inflammation control [152,153,154]. Diets rich in animal-based protein, especially red and processed meats, increase harmful metabolites like TMAO [155]. Ultra-processed foods, with additives, artificial sweeteners, and emulsifiers, damage the gut’s mucus layer, increase permeability, and promote endotoxemia, contributing to atherosclerosis and heightened MI risk [156]. High-fructose diets, commonly from sugary beverages and sweets, disrupt the gut microbiota, favoring pro-inflammatory bacteria [157]. High glycemic diets also increase CVD risk by causing oxidative stress, LDL oxidation, inflammation, protein glycation, and procoagulant activity [143,158]. These findings highlight the importance of reducing refined and processed foods and increasing fiber- and polyphenol-rich foods to support a heart-healthy gut microbiota and reduce MI risk.

#### 3.3.2. Sedentarism and Physical Inactivity

Physical inactivity comprises two independent but interrelated behaviors: insufficient moderate-to-vigorous physical activity and prolonged sedentary behavior. Epidemiological studies indicate that sedentary behavior is a significant predictor of CVD risk and a major contributor to the overall CVD burden [159]. Specifically, high sedentary time (>4 h/day) and physical inactivity are notably associated with all-cause mortality among MI survivors [160,161]. Sedentary lifestyles are also linked to alterations, including reduced microbial diversity and shifts in metabolic activity, resulting in lower network complexity than observed in active individuals [162,163,164,165,166]. In particular, sedentary behavior is associated with reduced levels of *Ruminococcaceae*, a butyrate-producing bacterial family, and increased levels of *Streptococcus* spp., especially in older adults [167]. This imbalance suggests a diminished capacity for butyrate production, a metabolite crucial for maintaining intestinal barrier integrity and anti-inflammatory effects. Further evidence from a hypoactivity model, such as dry immersion, demonstrated that certain bacteria and metabolites, including propionate, are sensitive to reduced physical inactivity, potentially impacting health during prolonged sedentary lifestyles [168]. Additionally, studies on sedentary women revealed higher abundances of bacteria linked to inflammatory profiles and adverse metabolic outcomes [169]. Recent research indicates that sedentary behavior and physical activity influence similar gut microbiota species in opposite directions; for instance, a sedentary lifestyle correlates with reduced carbohydrate-degrading capacity in the microbiota, particularly from dietary fibers, while physical activity supports a microbiome that fosters metabolic health [170,171]. These effects can be explained by both physiological and lifestyle factors. Physical inactivity may slow gut motility and reduce blood flow, creating a less favorable environment for diverse gut bacteria [172]. However, sedentary lifestyles often include unhealthy habits—like low fiber intake and high processed food consumption—that independently affect gut microbiota, making sedentarism a possible confounding variable. Clarifying these overlapping factors in research could help determine if inactivity alone changes the gut microbiome or if these changes are mostly due to associated behaviors. By targeting gut microbiota changes associated with sedentary behavior, interventions promoting physical activity could mitigate CVD risk and support overall cardiovascular health.

#### 3.3.3. Alcohol Consumption

Alcohol consumption is a known risk factor for multiple diseases, including CVD, but its relationship with MI risk varies based on drinking patterns and quantity [173,174]. For years, studies have suggested an association between moderate alcohol intake, particularly red wine, and improved heart health; however, recent evidence indicates that even a low level of alcohol consumption increases heart disease risk [175,176]. Episodes of heavy drinking, meanwhile, are strongly linked to an increased risk of MI, especially in older adults [177]. This elevated risk among heavy drinkers may be related to a pro-thrombotic state that arises during alcohol withdrawal phases, increasing the likelihood of ischemic events [178]. Beyond its cardiovascular effects, alcohol impacts the GI tract in ways that may exacerbate MI risk. Ethanol weakens the protective intestinal mucus layer, increasing gut permeability and promoting the overgrowth of Gram-negative bacteria that release inflammatory endotoxins, such as LPS [179,180,181]. This inflammation-induced vascular injury highlights the direct pathway by which alcohol-related gut dysbiosis and increased endotoxemia contribute to MI risk. Chronic alcohol use has also been shown to reduce gut microbiota diversity and promote dysbiosis, particularly in individuals with alcohol-associated liver disease or alcohol use disorder (AUD) [182,183]. Specifically, studies report an increase in Proteobacteria, a marker of gut dysbiosis, and a decrease in beneficial anaerobes like Bacteroidetes among heavy drinkers [181]. For patients with AUD, reducing alcohol intake and implementing microbiota-targeted therapies, such as probiotics, may help to rebalance gut health and reduce inflammation. These findings underscore the need for a nuanced approach to alcohol consumption, recognizing both the risks of heavy drinking and the emerging evidence that any alcohol consumption may increase cardiovascular risk.

## 4. Therapeutic Frontiers Targeting Gut Microbiota to MI Management

The gut–heart axis offers promising avenues for MI management, with dietary interventions, personalized probiotics, and exercise regimens emerging as key strategies to modulate gut microbiota and reduce cardiovascular risk. Table 2 provides a comprehensive summary of therapeutic strategies targeting the gut–heart axis, including specific mechanisms, evidence of benefits, population types studied, and key references for further reading.

### 4.1. Dietary Interventions

Dietary habits are the most important element in influencing both MI risk and gut microbiota composition. A meta-analysis of randomized controlled trials indicated that reducing dietary saturated fats leads to a 21% reduction in cardiovascular events [184]. Similarly, replacing saturated fatty acids with n-6 polyunsaturated fatty acids (PUFAs) has been associated with a 6% reduction in mortality in a prospective cohort followed over 16 years [185]. Rather than focusing on isolated components, improving overall dietary patterns appears to be a key driver in reducing all-cause and cardiovascular mortality post-MI [186].

#### 4.1.1. Mediterranean Diet (MD)

The Mediterranean diet (MD), characterized by a high intake of olive oil, fruits, nuts, vegetables, and whole grains, as well as a moderate intake of animal proteins and sugars, has shown substantial cardiovascular benefits [186,187]. High adherence to MD has been linked to lower all-cause mortality and a reduction in major cardiovascular events, including MI, stroke, and CVD mortality, in both primary and secondary prevention settings [188]. Compared to low-fat diets, MD has been associated with better outcomes in secondary prevention, likely due to its impact on various CVD risk factors, such as blood pressure, plasma cholesterol, arterial stiffness, and plaque stability [189,190,191,192,193,194,195]. The benefits of MD also extend to gut microbiota composition. Studies show that MD increases Bacteroidetes and decreases Firmicutes, lowering the Firmicutes/Bacteroidetes ratio, which is often linked to better metabolic outcomes [150,196]. MD has also been associated with an increase in SCFA-producing bacteria, which contribute anti-inflammatory properties and improve host metabolism [197,198,199]. Additionally, MD and other plant-based diets have been shown to lower levels of TMAO [200,201].

**Table 2:** Therapeutic approaches targeting gut microbiota for MI management.

Therapeutic Approach	Description	Mechanism	Evidence of Cardiovascular Benefit	References
Mediterranean Diet	High intake of olive oil, fruits, vegetables, nuts, and moderate animal protein	Increases SCFA-producing bacteria and lowers TMAO levels	Lowers MI risk and improves cardiovascular outcomes	[150,186,187,189,190,191,192,193,194,195,196,200,201]
Dietary Fiber	Rich in fruits, vegetables, and whole grains	Supports SCFA production and strengthens gut barrier integrity	Reduces systemic inflammation, endotoxemia, and MI risk	[26,202,203,204,205,206,207,208,209,210,211,212,213,214,215,216]
Antioxidants and Polyphenols	Found in foods like berries, tea, and olive oil	Act as prebiotics and promote beneficial bacteria	Decrease oxidative stress, support endothelial function, and lower MI risk	[145,217,218,219,220,221,222,223,224,225,226,227,228]
Personalized Probiotics	Administration of specific beneficial bacteria strains	Modulate gut microbiota and reduce inflammation	Reduce MI size and improve cardiac function in animal models	[229,230,231,232,233,234,235,236]
Physical Activity	Regular moderate-to-vigorous exercise	Enhances gut microbiota diversity and reduces inflammation	Associated with reduced CVD and all-cause mortality	[162,166,237,238,239,240,241,242,243,244,245,246,247,248]

#### 4.1.2. Fiber Intake

Dietary fiber intake is strongly associated with improved cardiovascular health and a reduced risk of MI [202,203,204,205,206,207]. Fiber, primarily found in fruits, vegetables, whole grains, and legumes, plays a critical role in supporting a healthy gut microbiota. Studies show that fiber intake promotes shifts in gut microbiome composition, increasing the abundance of beneficial bacteria like *Faecalibacterium prausnitzii*, *Bifidobacterium*, and *Lactobacillus*, while lowering the Firmicutes/Bacteroidetes ratio, a marker often associated with metabolic health improvements [208]. Fiber serves as a substrate for beneficial gut bacteria, especially those that produce SCFAs, for maintaining intestinal barrier integrity, as they strengthen tight junctions between epithelial cells, reduce gut permeability, and protect against the translocation of endotoxins to the bloodstream [209]. These effects can significantly impact systemic inflammation, lipid metabolism, and vascular health, which are all critical factors in MI risk [210,211]. In particular, butyrate and propionate produced by commensal bacteria such as *Lactobacillus* and *Bifidobacterium* have been shown to reduce inflammation and strengthen the gut barrier. This offers protective effects against cardiac damage by inhibiting immune cell activation and reducing systemic inflammatory markers, including IL-6, TNF-alpha-R2, and C-reactive protein (CRP) [212,213]. High-fiber diets have also been seen to decrease endotoxemia by fostering a microbiota composition that discourages the growth of LPS-producing bacteria [249]. Moreover, dietary fiber supplementation is linked to reduced activation of macrophages and dendritic cells, and it enhances the suppressive function of T-regulatory cells, thereby reducing metabolic endotoxemia and inflammatory responses [214,215]. In animal studies, high-fiber diets have been shown to reduce infarct size and improve cardiac function post-MI, likely due to the protective role of SCFAs on cardiomyocytes and their immune-modulating effects [26,209,216]. These findings underscore the importance of dietary fiber as a cornerstone of dietary interventions aimed at reducing MI risk and supporting overall cardiovascular health.

#### 4.1.3. Antioxidants and Polyphenols

The role of dietary antioxidants and polyphenols in cardiovascular health, particularly for managing MI, has gained significant attention. These compounds, abundant in fruits, vegetables, tea, coffee, nuts, olive oil, and dark chocolate, offer protective effects on cardiovascular health through their impact on systemic inflammation, endothelial function, and gut microbiota [145]. Unlike other nutrients, polyphenols are not fully absorbed in the upper GI tract; instead, they reach the colon, where they interact with gut microbiota, acting as prebiotic substrates to support the growth of beneficial bacteria such as *Lactobacillus* and *Bifidobacterium*, while reducing the number of pathogenic bacteria such as *Escherichia coli*, *Clostridium perfringens*, and *Helicobacter pylori* [217,218]. Through microbial metabolism, polyphenols are converted into bioactive metabolites, such as urolithins and phenolic acids, which exert anti-inflammatory and antioxidant effects crucial for cardiovascular health [219,220]. For instance, anthocyanins and resveratrol found in berries and grapes are known to increase SCFA production, inhibit pathogenic bacteria, and improve endothelial function by reduce oxidative stress [221,222,223]. These effects may contribute to lower plaque formation risk and improved plaque stability, both essential in MI prevention and management [224,225]. Green tea and coffee contain catechins and chlorogenic acid, respectively, which support beneficial gut microbiota, reduce endotoxemia, and improve blood pressure and cholesterol levels, key factors in cardiovascular health [226,227]. Extra virgin olive oil, a staple in the MD, is rich in hydroxytyrosol and oleuropein, which possess potent anti-inflammatory and antioxidant effects that foster a gut environment favorable beneficial bacteria, thereby reducing oxidative stress and systemic inflammation [228]. These evidence linking dietary antioxidants and polyphenols to improved cardiovascular outcomes via gut microbiota modulation underscore the therapeutic potential of these compounds in MI management. Incorporating polyphenol-rich foods into dietary interventions offers a natural, multifaceted approach to enhance gut health, reduce inflammation, and protect against MI. Further research, including randomized clinical trials, is needed to clarify optimal dosages, effective polyphenol sources, and their long-term effects on cardiovascular health and MI prevention.

### 4.2. Personalized Probiotics

Probiotics are living microorganisms that can be ingested for their potential health benefits, including enhancing the immune microenvironment and increasing microbial richness and diversity. Studies have demonstrated that certain bacterial strains, when administered based on a patient’s microbiome, can improve cardiac outcomes by reducing systemic inflammation and oxidative stress, both critical in post-MI recovery [229]. For example, many animal studies have confirmed that probiotics, as a combination of *Lactobacillus acidophilus* and *Bifidobacterium animalis* subsp. *lactis*, reduced myocardial infarct size in rats with diet-induced obesity and chemically-induced colitis, suggesting their applicability in ischemic heart disease prevention [232]. Similarly, supplementation with strains like *Lactobacillus rhamnosus* and *Lactobacillus johnsonii* has been shown to reduce the MI size, cardiac hypertrophy, and improve cardiac function after MI by reprogramming the gut microbiome [231,233,236]. Interestingly, a randomized, placebo-controlled clinical trial evaluating the effects of *Lactobacillus rhamnosus* supplementation on symptoms of depression and quality of life in MI patients reported significant improvements in depressive symptoms, quality of life, and markers of oxidative stress and inflammation [230]. Despite traditional probiotic treatments have often utilized broad-spectrum bacterial strains, such as *Lactobacillus* and *Bifidobacterium*, to restore microbial balance and enhance gut barrier integrity; emerging evidence suggests that personalized probiotics tailored to an individual’s unique microbiota composition may provide superior benefits in the context of MI recovery, playing a vital role in advancing treatment approaches [232]. For instance, a clinical trial reported that *Saccharomyces boulardii* supplementation reduced ventricular remodeling after MI and improved ventricular systolic function in patients with HE, indicating potential benefits for MI prognosis [250]. Additionally, a recent study explored the effect of daily oral administration of an engineered probiotic derived from a modified *Escherichia coli* Nissle 1917 strain, designed to continuously secrete SCFAs as a preventive strategy against myocardial injury in an I/R animal model [251]. This intervention significantly reduced myocardial injury and improved cardiac performance compared to controls receiving the unmodified strain, likely due to reduced neutrophil infiltration into the infarct site and promotion of wound-healing macrophage polarization. These findings highlight the potential of tailored probiotic therapies as a natural, multifaceted approach to supporting cardiovascular health post-MI. However, further research, including human clinical trials, is needed to optimize strain selection, dosing, and therapeutic timing.

### 4.3. Exercise Regimens

Physical activity, which increases energy expenditure, is widely recognized as a critical approach to managing multiple conditions linked to CVD and MI risk [237]. Current guidelines recommend that adults engage in at least 150 min of moderate-intensity aerobic exercise or a minimum of 75 min of vigorous-intensity aerobic exercise weekly to prevent CVD [238]. Adherence to these recommendations has been shown to reduce CVD and all-cause mortality by 23–40% and 27–30%, respectively [239,240]. In secondary prevention, regular exercise training is essential for therapeutic intervention, with exercise adherence linked to a reduction in adverse outcomes over a 36-month follow-up in patients with stable heart disease [241]. Replacing just 30 min per day of sedentary behavior with physical activity is associated with a 3–12% reduction in CVD risk, further emphasizing the cardioprotective effects of exercise [242]. These benefits are mediated through multiple mechanisms, including improvement of insulin resistance, hypertension, dyslipidemia, endothelial dysfunction, and systemic inflammation [237,243]. However, a prospective study in healthy middle-aged women revealed that traditional CVD risk factors accounted for only 59% of the observed reduction in CVD risk due to exercise, suggesting that other less conventional factors play a role in these cardioprotective effects [244]. Emerging evidence indicates that exercise independently alters the composition and functional capacity of the gut microbiota, which may contribute to these cardiovascular benefits [245]. Cross-sectional studies in humans report that professional athletes have greater gut microbiota diversity, with a higher abundance of Firmicutes and a lower abundance of Bacteroidetes compared to lean sedentary controls [166,246]. However, studies in older adults have shown inconsistent results, with some reporting no associations between physical activity and gut microbiota diversity [162,247,248]. In this age group, other factors like weight and biological age may also influence the microbiota response to exercise, indicating a need for further research to clarify these interactions. In a recent study, an eight-week exercise regimen after left anterior descending coronary artery ligation in animal models altered gut microbiota composition, increasing Bacteroidetes and decreasing Firmicutes compared to non-exercising controls [70]. These findings suggest that the positive effects of exercise on gut microbiota may partially explain the cardiovascular improvements seen in individuals with active lifestyles. Thus, enhancing gut microbiota composition through exercise could be considered a supplementary therapeutic approach for MI management. Nevertheless, further studies are needed to determine the optimal type, intensity, and duration of exercise to achieve the best results for both gut health and cardiovascular protection.

## 5. Challenges and Future Directions

Next-generation sequencing has significantly advanced our understanding of the human gut microbiota by enabling the discovery and characterization of unculturable microbes and predicting their functions [44]. Although the gut–heart axis holds promise for MI treatment, research and clinical application face challenges due to the complexity of gut microbiota composition, which is influenced by genetics, sex, aging, lifestyle, and environmental factors [252]. While our discussion focuses primarily on the impact of lifestyle habits such as diet, physical inactivity, and alcohol consumption on the gut–heart axis, other lifestyle factors—such as pollutants, stress, and sleep quality—also play significant roles. These factors contribute to gut dysbiosis and subsequent inflammation, potentially exacerbating MI risk [253]. Although not the primary focus of our review, these interconnected factors interact with diet, physical activity, and alcohol intake to influence the gut–heart axis. Addressing these factors holistically could enhance cardiovascular disease prevention by promoting a healthier gut microbiome and reducing inflammation.

Investigating the gut–heart axis and its role in MI remains challenging due to inconsistent data across diverse populations. These discrepancies are often attributed to differences in diet, ethnicity, lifestyle, environmental exposures, and research methodologies, all of which affect gut microbiota composition and its cardiovascular impact [254,255]. Diet, in particular, is a key determinant of microbiota composition and varies widely across region and culture, complicating the synthesis of global conclusions. Ethnic differences in microbiota profiles can also lead to varying susceptibility to MI, making it challenging to generalize findings. Furthermore, methodological variations—such as differences in sample collection, sequencing techniques, and data analysis—further complicate cross-study comparisons. To address these issues, large-scale, multi-ethnic cohort studies and standardized methods are needed. Advances in sequencing and multi-omics (genomics, metabolomics, and proteomics) could help identify specific microbial functions related to the gut–heart axis, enabling more tailored interventions. Developing reliable biomarkers, such as specific microbial profiles or metabolites, is crucial for personalized treatments and monitoring MI recovery. A deeper understanding of these complexities will improve the precision of gut microbiota-based therapies for MI prevention and treatment.

An important area for future research involves exploring the bidirectional nature of the gut–heart relationship. While gut dysbiosis can affect MI outcomes, the impact of MI and related treatments on gut health, particularly under conditions of stress or medication, remains underexplored. Long-term prospective studies in MI patients are needed to clarify how changes in gut microbiota influence cardiac function over time and, conversely, how cardiac injury alters gut microbiota composition. Leveraging multi-omics techniques can provide a comprehensive view of the molecular interactions between the gut and the heart. Additionally, mechanistic studies using animal models that mimic human MI conditions, such as the SR-B1^−/−^ApoE-R61^h/h^ model, are essential for dissecting the pathways involved in gut–heart interactions. These models can help explore how gut permeability, endotoxemia, and specific microbial metabolites contribute to cardiac damage, as well as how cardiac dysfunction influences gut health.

## 6. Conclusions

The significant changes in Western dietary patterns over the past 50 years have brought various benefits, such as reductions in malnutrition and gastrointestinal infections. However, this rapid dietary transformation has also affected multiple bodily systems, notably the intestinal microbiota. Although the precise functional role of individual microbiota components remains incompletely understood, growing evidence points to a symbiotic relationship between the host and its microbes. The gut–heart axis has thus emerged as a crucial factor in understanding and managing MI. Disruptions in gut integrity and microbial balance, along with the influence of gut-derived metabolites like LPS and TMAO, significantly exacerbate systemic inflammation and cardiovascular injury in MI contexts. Conversely, beneficial metabolites such as SCFAs provide protective effects, highlighting the potential of gut-targeted interventions—including dietary modifications, personalized probiotics, antioxidants, polyphenols, and exercise—as promising therapeutic strategies. While research supports the efficacy of these interventions, advancing the field requires overcoming challenges related to biomarker development, interindividual variability, and long-term clinical outcomes. Integrating the gut–heart axis into MI management could lead to more holistic and effective treatment approaches, ultimately reducing CVD burden and improving patient recovery. However, due to limited evidence on causality in human subjects, the precise sequence of pathological events between gut microbiota alterations and host status remains a “chicken-and-egg” dilemma. 

## Figures and Tables

**Table 1:** Main biomarkers of intestinal barrier dysfunction in MI.

Biomarker	Population	Technique	Change in MI	References
ZO	MI patients	Serum biomarker analysis	Elevated ZO levels correlated with systemic inflammation	[20,21,22,23]
I-FABP	MI patients	Serum biomarker analysis	Higher I-FABP levels linked to larger infarct sizes and worsened cardiac function	[24]
LPS	MI patients and experimental MI models	Serum biomarker analysis	Increased serum LPS triggering systemic inflammation via TLR4 activation, worsening myocardial damage	[20,21,22,23,25,26]
D-lactate	MI patients and experimental MI models	Serum biomarker analysis	Elevated D-lactate levels associated with systemic inflammation and predictive of adverse cardiovascular events	[26]
